# Mechanistic Insights Into Acute Kidney Injury Complicating Invasive Pulmonary Aspergillosis in Adults

**DOI:** 10.7759/cureus.114372

**Published:** 2026-08-11

**Authors:** Xiaolong Li, Xin Wang, Rongli Wang, Yinhe Feng, Chao Qin, Yubing Yue, Shuhao Xu

**Affiliations:** 1 Pulmonary and Critical Care Medicine, Deyang People's Hospital, Deyang, CHN; 2 Pulmonary and Critical Care Medicine, The Affiliated Hospital of Southwest Medical University, Luzhou, CHN; 3 Stomatology, Deyang People's Hospital, Deyang, CHN

**Keywords:** acute kidney injury, drug nephrotoxicity, fungal toxins, inflammatory response, invasive pulmonary aspergillosis, mechanisms

## Abstract

Invasive pulmonary aspergillosis (IPA) is a life-threatening fungal infection predominantly affecting immunocompromised patients, with a rising incidence observed in atypical hosts such as critically ill individuals. Acute kidney injury (AKI) represents a severe and frequent complication of IPA, markedly increasing morbidity and mortality rates. Despite its clinical significance, the pathophysiological mechanisms underlying IPA-associated AKI remain incompletely understood. This review provides a comprehensive examination of the multifactorial processes contributing to AKI in the context of IPA, including the direct nephrotoxic effects of fungal toxins, dysregulated host immune and inflammatory responses, drug-induced renal toxicity, and hemodynamic disturbances. By integrating recent advances from molecular biology, immunology, and clinical studies, we aim to elucidate the complex interplay of factors driving renal impairment in IPA patients. Understanding these mechanisms is crucial for the development of early diagnostic markers and targeted therapeutic strategies to improve patient outcomes. This comprehensive overview provides a theoretical foundation for future research and clinical management of IPA complicated by AKI.

## Introduction and background

Invasive pulmonary aspergillosis (IPA) is a severe and often life-threatening fungal infection primarily caused by *Aspergillus* species, most notably *Aspergillus fumigatus*. It predominantly affects immunocompromised individuals, including those with prolonged neutropenia, hematopoietic stem cell or solid organ transplantation, inherited or acquired immunodeficiencies, and patients receiving corticosteroids or other immunosuppressive therapies. The clinical presentation of IPA encompasses a broad spectrum, ranging from subacute to critically severe invasive disease, and carries a considerable mortality risk, which is particularly evident in the intensive care unit (ICU) setting. Relevant studies have demonstrated that the 90-day mortality rate of IPA in the ICU is as high as 33.3% [1,2]. The pathogenesis of IPA involves the inhalation of airborne *Aspergillus conidia*, which can evade host immune defenses in susceptible hosts, leading to tissue invasion, angioinvasion, and subsequent dissemination. The diagnosis of IPA remains challenging due to nonspecific clinical manifestations and the limitations of current diagnostic modalities, which include radiological imaging, microbiological cultures, histopathology, and fungal biomarkers such as galactomannan and (1→3)-β-D-glucan assays. Early and accurate diagnosis is critical for initiating timely antifungal therapy, which significantly improves patient outcomes [3,4].

The epidemiology of IPA has evolved over recent years, with an increasing incidence observed not only in classical immunocompromised populations but also among non-neutropenic patients, including those with chronic obstructive pulmonary disease (COPD), severe viral pneumonias such as COVID-19 and influenza, and critically ill patients in ICUs. The COVID-19 pandemic has notably highlighted the emergence of COVID-19-associated pulmonary aspergillosis (CAPA), which complicates the clinical course of severe COVID-19 and is associated with increased mortality. Similarly, influenza-associated IPA has been recognized as a significant contributor to respiratory failure and death in critically ill patients [5-7]. These observations underscore the expanding at-risk populations and the need for heightened clinical vigilance and refined diagnostic criteria tailored to diverse patient groups. Moreover, the increasing use of immunosuppressive agents, including corticosteroids, monoclonal antibodies, and novel anticancer drugs, further predisposes patients to IPA by impairing innate and adaptive immune responses.

Among the extrapulmonary complications of IPA, acute kidney injury (AKI) has emerged as a frequent and clinically significant event, occurring in approximately 20% to 52% of IPA patients [8,9]. AKI in this context is associated with prolonged hospitalization and increased mortality, representing a critical determinant of patient prognosis. Traditionally, AKI in IPA patients has been attributed mainly to the nephrotoxic effects of antifungal agents, particularly amphotericin B formulations, which remain cornerstone therapies for invasive fungal infections. However, recent studies have elucidated that the pathogenesis of AKI in IPA is multifactorial, involving not only drug-induced nephrotoxicity but also direct fungal effects and host immune-mediated mechanisms. For instance, fungal toxins induced oxidative stress, leading to cell death by the intrinsic pathway of apoptosis in renal cells [10]. Concurrently, the host’s inflammatory response, characterized by cytokine release and immune cell infiltration, may exacerbate renal damage through systemic inflammatory pathways [11]. These insights highlight the complexity of IPA-associated AKI and the necessity for comprehensive mechanistic studies to inform targeted therapeutic strategies.

Given the intricate interplay of fungal virulence factors, host immune responses, pharmacological effects, and hemodynamic alterations in the development of AKI during IPA, a multidimensional investigative approach is warranted. This review aims to synthesize current knowledge on the mechanisms underlying AKI in adult patients with IPA, focusing on four key dimensions: fungal toxins and angioinvasion, immune-inflammatory responses, antifungal drug-related nephrotoxicity, and hemodynamic disturbances. By integrating recent clinical and experimental findings, we seek to elucidate the pathophysiological pathways contributing to renal injury in IPA [12,13]. Furthermore, we discuss emerging diagnostic tools and antifungal agents that may mitigate renal complications while effectively controlling fungal infection. Ultimately, advancing our understanding of IPA-associated AKI will enhance clinical management and improve outcomes for this vulnerable patient population.

## Review

Direct nephrotoxic mechanisms of fungal toxins

Toxic Effects of Fungal Toxins on Renal Vascular Endothelial Cells

Fungal toxins produced by *Aspergillus* species also target the renal microvasculature, particularly the glomerular capillary endothelial cells, disrupting the integrity of the vascular barrier. This endothelial injury manifests as increased permeability, leading to proteinuria and a decline in glomerular filtration rate (GFR). Animal models have provided compelling evidence that intravenous administration of fungal toxins results in microvascular thrombosis and endothelial cell swelling within renal tissues, indicating a direct toxic effect on the vascular endothelium. The endothelial damage is further exacerbated by the upregulation of adhesion molecules such as intercellular adhesion molecule-1 (ICAM-1) and vascular cell adhesion molecule-1 (VCAM-1), which facilitate leukocyte adhesion and transmigration into renal tissues. This leukocyte infiltration amplifies local inflammatory responses, contributing to endothelial dysfunction and microvascular injury. The resultant microvascular thrombosis and inflammation impair renal perfusion and filtration, thereby aggravating renal injury. These findings elucidate the critical role of fungal toxin-induced endothelial toxicity in the pathophysiology of AKI associated with IPA, emphasizing the importance of vascular injury in fungal nephrotoxicity [14,15].

Promotion of Renal Interstitial Fibrosis by Fungal Toxins

**Figure 1:**
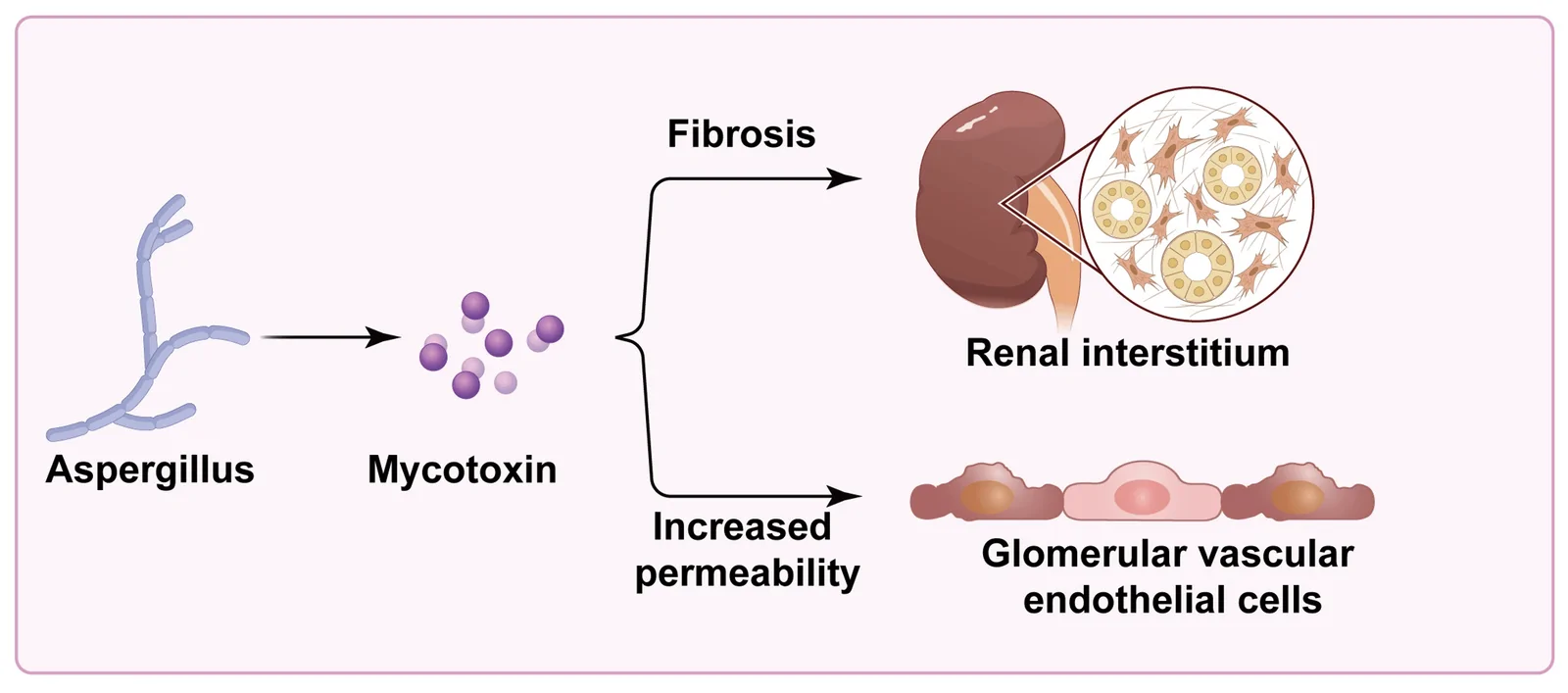
Fungal toxin-mediated nephrotoxicity Fungal toxins also damage glomerular vascular endothelial cells, compromising vascular barrier integrity, and promote renal interstitial fibrosis via the TGF-β1/Smad pathway. This figure was made using Adobe Illustrator (Adobe Inc., San Jose, CA, USA).

Chronic exposure to low concentrations of *Aspergillus*-derived mycotoxins has been implicated in the activation of renal interstitial fibroblasts, promoting fibrosis through the TGF-β1/Smad signaling pathway. This profibrotic cascade leads to excessive collagen deposition within the renal interstitium, contributing to the progression of chronic kidney damage. Fungal toxin-induced oxidative stress upregulates matrix metalloproteinases (MMP-2 and MMP-9), enzymes that degrade the tubular basement membrane, facilitating fibroblast activation and extracellular matrix remodeling. Clinical pathological analyses of renal biopsy specimens from patients with IPA complicated by AKI reveal focal interstitial fibrosis, with the extent of fibrosis correlating positively with the duration of fungal toxin exposure. This fibrotic remodeling impairs renal architecture and function, potentially leading to irreversible kidney injury. The interplay between oxidative stress, MMP activation, and TGF-β1 signaling underscores a multifaceted mechanism by which fungal toxins contribute to renal fibrosis in IPA-associated AKI. Understanding these pathways offers potential therapeutic targets to mitigate fibrosis and preserve renal function in affected patients (Figure 1) [16,17].

Role of host immune-inflammatory dysregulation in AKI

Excessive Inflammatory Response and Cytokine Storm

IPA triggers a robust activation of the host innate immune system, primarily involving macrophages and neutrophils that respond to fungal invasion by releasing a plethora of pro-inflammatory cytokines such as tumor necrosis factor-alpha (TNF-α), interleukin-1 beta (IL-1β), and interleukin-6 (IL-6). This cytokine surge can escalate into a systemic inflammatory response syndrome (SIRS), which is a hallmark of severe IPA and contributes significantly to organ dysfunction, including AKI. Elevated circulating TNF-α levels have been shown to directly injure renal tubular epithelial cells by binding to the TNF receptor 1 (TNFR1), thereby activating the nuclear factor kappa B (NF-κB) signaling pathway. This activation induces the transcription of inflammatory genes and promotes apoptosis of tubular cells, exacerbating renal damage. IL-6 further amplifies renal inflammation by engaging the gp130/STAT3 signaling cascade in tubular cells, leading to the upregulation of chemokines such as monocyte chemoattractant protein-1 (MCP-1). MCP-1 recruits additional inflammatory cells into the renal interstitium, perpetuating a cycle of inflammation and injury [12,18]. Clinical observations in IPA patients with AKI support this mechanistic framework, where systemic cytokine elevations correlate with the severity of renal impairment. Moreover, the cytokine storm not only damages renal parenchyma but also disrupts microvascular perfusion, contributing to ischemic injury. The interplay between fungal pathogen-associated molecular patterns (PAMPs) and host pattern recognition receptors (PRRs) initiates this inflammatory cascade, but dysregulation and overactivation of these responses lead to collateral tissue damage. Targeting inflammatory pathways may have theoretical benefit in reducing AKI in IPA, but such approaches must be carefully balanced against the risk of impairing antifungal immunity and potentially exacerbating fungal infection. Understanding the balance between effective antifungal immunity and excessive inflammation is critical, as immunosuppression to control inflammation may risk exacerbating fungal proliferation. This clinical dilemma is particularly pronounced in multimorbid patients with pre-existing conditions like chronic kidney disease and diabetes, where immunomodulatory therapies can complicate the diagnosis and management of disease [19]. Thus, the excessive inflammatory response and cytokine storm represent a pivotal mechanism by which IPA induces AKI, highlighting the need for precise modulation of host immunity to prevent renal injury while controlling fungal infection [20,21].

Neutrophil Extracellular Traps (NETs) and Renal Injury

During IPA, neutrophils deploy neutrophil extracellular traps (NETs) as a defense mechanism to ensnare and neutralize *Aspergillus hyphae* and *conidia*. NETs consist of a web-like structure of decondensed chromatin decorated with antimicrobial proteins, including histones, myeloperoxidase (MPO), and proteases. While NET formation is crucial for fungal containment, excessive or dysregulated NET release can inflict collateral damage on host tissues, notably the renal tubular epithelium. Histones H3 and H4, abundant in NETs, have cytotoxic properties; they can bind directly to tubular epithelial cell membranes, triggering calcium influx that leads to necrotic cell death [22,23]. Additionally, these histones activate the complement cascade, particularly the alternative pathway, resulting in further inflammation and tissue injury. MPO and proteases released within NETs degrade extracellular matrix components and disrupt cellular integrity, exacerbating tubular damage. Clinical studies have demonstrated elevated plasma levels of NET markers, such as citrullinated histone H3, in IPA patients complicated by AKI, with levels correlating positively with the degree of renal dysfunction [12]. This suggests that NET-mediated injury is a significant contributor to AKI pathogenesis in IPA. The dual role of NETs as both antifungal effectors and mediators of tissue injury underscores the complexity of host-pathogen interactions in IPA. Therapeutic strategies aimed at modulating NET formation or promoting NET clearance, such as DNase administration, may offer renal protection but require careful evaluation to avoid impairing antifungal defense. The recognition of NETs’ pathogenic role in IPA-associated AKI expands the understanding of immune-mediated renal injury and identifies novel targets for intervention.

Complement System Overactivation and Tubular Injury

**Figure 2:**
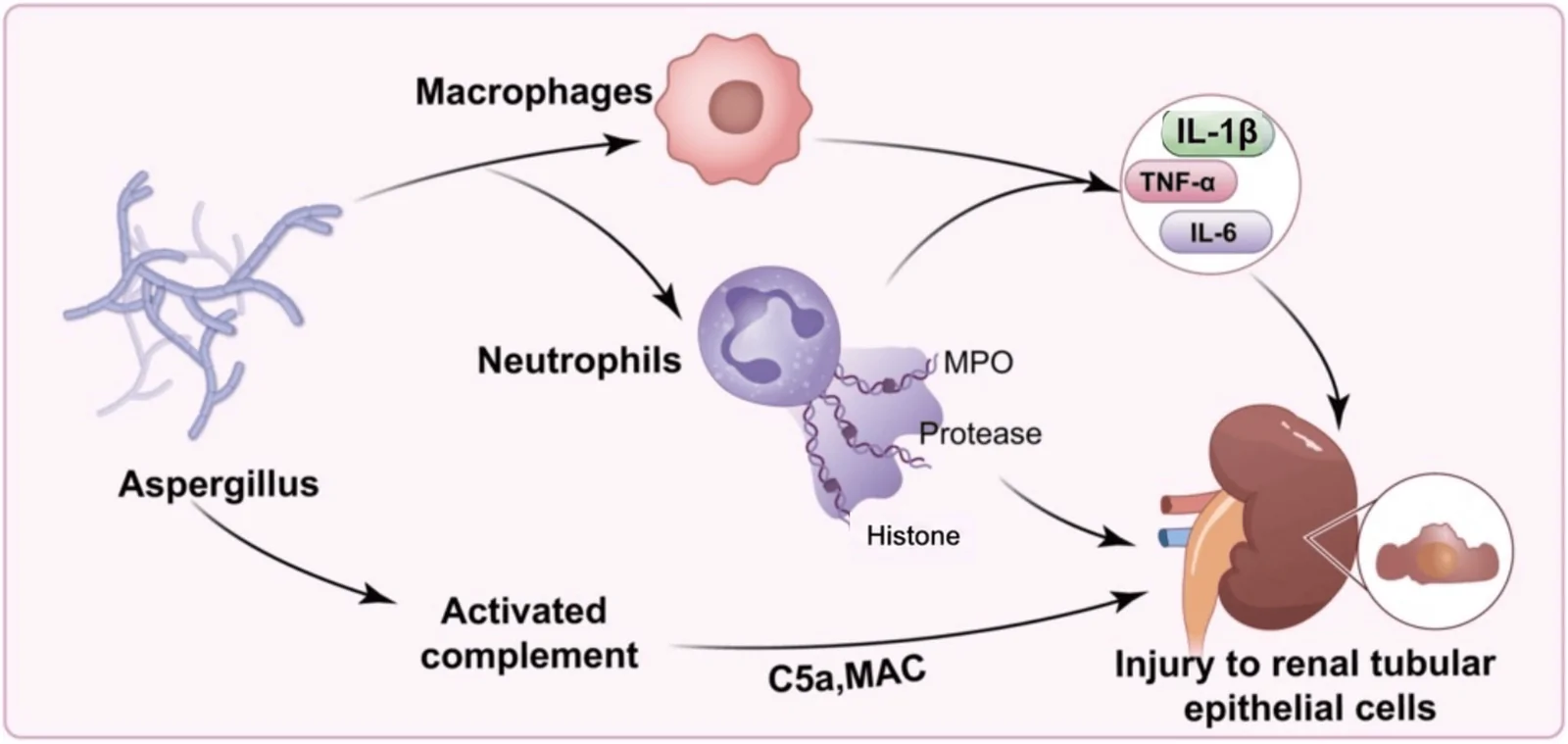
Immune-mediated renal injury *Aspergillus* activates macrophages and neutrophils, leading to excessive release of pro-inflammatory cytokines (TNF-α, IL-1β, and IL-6) that damage tubular cells. NETs released to contain fungal elements cause direct tubular injury through their histone, myeloperoxidase, and protease components. Complement activation with the generation of C5a and MAC further enhances tubular apoptosis. NET: neutrophil extracellular trap; MAC: membrane attack complex This figure was made using Adobe Illustrator (Adobe Inc., San Jose, CA, USA).

The complement system, a key component of innate immunity, is excessively activated during IPA, primarily through the alternative pathway triggered by fungal cell wall components such as β-glucans. This activation leads to the generation of potent inflammatory mediators, including C5a and the membrane attack complex (MAC). C5a binds to its receptor C5aR1 expressed on renal tubular epithelial cells, inducing reactive oxygen species (ROS) production and mitochondrial dysfunction, which promote apoptosis and impair cellular metabolism. Concurrently, deposition of MAC on the tubular basement membrane compromises membrane integrity, causing cell lysis and necrosis. This complement-mediated cytotoxicity contributes significantly to tubular injury and the progression of AKI in IPA. Experimental and clinical data support this mechanism; elevated levels of complement activation products have been detected in patients with IPA and AKI, correlating with worse renal outcomes [22,24,25]. The complement cascade thus acts as a double-edged sword, facilitating fungal clearance but also mediating host tissue damage when overactivated. Targeting complement components, such as C5a or MAC formation, represents a promising therapeutic avenue to attenuate renal injury without compromising antifungal immunity. However, the challenge lies in achieving selective inhibition to preserve the protective functions of complement. Understanding the precise role of complement overactivation in IPA-associated AKI is essential for developing adjunctive therapies that mitigate renal damage while maintaining effective host defense (Figure 2).

Mechanism of antifungal drug-related renal injury

Nephrotoxic Mechanisms of Amphotericin B

Amphotericin B (AmB), a polyene antifungal agent, remains a cornerstone in the treatment of invasive fungal infections such as IPA, despite its well-documented nephrotoxicity. The nephrotoxic mechanisms of AmB are multifactorial and primarily involve direct tubular cell injury and renal hemodynamic alterations. At the cellular level, AmB binds to ergosterol-like sterols in the membranes of renal tubular epithelial cells, particularly in the distal tubules, forming transmembrane ion channels that disrupt membrane integrity. This pore formation leads to leakage of intracellular potassium ions and cellular depolarization, culminating in tubular cell injury and death. The resultant cell damage compromises tubular function and contributes to AKI [26]. Beyond direct cytotoxicity, AmB induces renal vasoconstriction, especially of the afferent arterioles, which decreases renal blood flow and GFR. This vasoconstriction is mediated by increased release of vasoconstrictive agents such as endothelin and decreased production of vasodilators like nitric oxide, exacerbating ischemic injury to the renal parenchyma [27]. Furthermore, AmB activates NADPH oxidase enzymes within renal tubular cells, leading to excessive generation of ROS, particularly superoxide anions. This oxidative stress triggers lipid peroxidation, mitochondrial dysfunction, and activation of pro-apoptotic signaling pathways, amplifying tubular injury and inflammation [15]. The oxidative damage also promotes interstitial fibrosis if the injury becomes chronic. Clinically, these mechanisms manifest as a decline in renal function, electrolyte disturbances (notably hypokalemia and hypomagnesemia), and, in severe cases, AKI requiring renal replacement therapy. Lipid formulations of AmB, such as liposomal AmB, have been developed to reduce nephrotoxicity by altering drug distribution and reducing free drug exposure to renal tubular cells; however, nephrotoxicity remains a significant concern, especially at higher cumulative doses. Recent pharmacovigilance data confirm that AmB exhibits the strongest renal toxicity signals among antifungal agents, with high rates of nephropathy and renal tubular disorders correlating with increased mortality [16]. Despite these risks, AmB’s broad antifungal spectrum and efficacy in refractory cases justify its use, necessitating careful monitoring of renal function and judicious dosing. Novel polyenes with enhanced ergosterol selectivity and reduced nephrotoxicity, such as EL219, are under investigation and show promise in preclinical models by improving survival and reducing fungal burden with less renal toxicity [28].

Nephrotoxic Risks of Azole Antifungals

Azole antifungals, including voriconazole and itraconazole, are widely used triazoles for the treatment of invasive fungal infections such as IPA. While generally considered less nephrotoxic than polyenes, azoles carry distinct renal risks primarily related to their pharmacokinetic properties and excipient formulations. Voriconazole exerts its antifungal effect by inhibiting fungal cytochrome P450 (CYP450) enzymes, but it also inhibits human CYP450 isoforms involved in drug metabolism. This inhibition can disrupt renal tubular cell metabolism, leading to intracellular accumulation of toxic metabolites and subsequent cellular injury. Additionally, voriconazole’s metabolism is highly variable and influenced by genetic polymorphisms, drug interactions, and organ dysfunction, which can result in supratherapeutic levels and increased toxicity [28,29]. Therapeutic drug monitoring is therefore essential to balance efficacy and toxicity. Itraconazole formulations often contain cyclodextrin excipients such as hydroxypropyl-β-cyclodextrin to enhance solubility. These excipients are renally cleared and can accumulate in patients with impaired renal function, exacerbating tubular toxicity and contributing to nephrotoxicity [16]. Chronic azole exposure has been associated with tubulointerstitial nephritis and renal fibrosis, characterized histologically by interstitial inflammation and scarring, which clinically manifests as a gradual rise in serum creatinine and proteinuria. This chronic injury likely results from persistent low-grade inflammation and oxidative stress induced by azole metabolites or excipients [30]. Moreover, azoles can cause indirect renal injury through drug-drug interactions that increase nephrotoxic potential of concomitant medications, such as calcineurin inhibitors in transplant recipients. Clinical reports highlight cases of AKI during voriconazole therapy, especially in critically ill patients or those receiving extracorporeal membrane oxygenation (ECMO), where voriconazole sequestration in circuits leads to subtherapeutic dosing and necessitates dose escalation, increasing toxicity risk [31,32]. Despite these risks, azoles remain first-line agents due to their oral bioavailability and broad antifungal spectrum. Newer azoles like isavuconazole offer improved safety profiles with less hepatotoxicity and nephrotoxicity and delayed adverse event onset, making them attractive alternatives [33]. In conclusion, azole nephrotoxicity arises from CYP450-mediated metabolic disruption, excipient accumulation in renal impairment, and chronic tubulointerstitial inflammation. Careful dosing, therapeutic drug monitoring, and consideration of renal function are essential to minimize renal adverse effects during azole therapy.

Echinocandins: Renal Safety and Potential Risks

Echinocandins, including caspofungin, micafungin, and anidulafungin, represent a class of antifungal agents that inhibit fungal β-(1,3)-D-glucan synthesis, disrupting cell wall integrity. These agents are predominantly metabolized hepatically, with minimal renal excretion, which confers a relatively low nephrotoxic risk compared to polyenes and azoles. Clinical pharmacovigilance data corroborate the lower incidence of renal adverse events with echinocandins, although hepatotoxicity remains a concern [33]. However, high-dose echinocandin administration may still induce renal tubular cell stress, as suggested by preclinical studies, indicating a dose-dependent potential for nephrotoxicity under certain conditions [34]. Micafungin, in particular, has demonstrated renal protective effects in animal models by inhibiting tubular cell apoptosis, potentially through modulation of oxidative stress pathways, although clinical evidence remains limited and warrants further investigation [20]. Importantly, the risk of AKI may be amplified during combination antifungal therapy, especially when echinocandins are co-administered with other nephrotoxic agents such as aminoglycosides or AmB. Such drug interactions can potentiate tubular injury and complicate clinical management. Despite their favorable renal safety profile, echinocandins are not available orally and are typically reserved for intravenous use, limiting their utility in long-term outpatient therapy. Emerging echinocandin derivatives with improved pharmacokinetics, such as rezafungin with once-weekly dosing, and oral agents like ibrexafungerp, hold promise for expanding therapeutic options while maintaining safety [35]. In clinical practice, echinocandins are often employed in combination regimens or as salvage therapy for invasive aspergillosis, particularly in patients with renal impairment, where azoles or polyenes pose higher risks. Close monitoring remains prudent, especially in critically ill patients receiving multiple nephrotoxic drugs.

Hemodynamic disorders and insufficient renal perfusion

Hemodynamic Mechanisms of Sepsis-Associated AKI

IPA can precipitate systemic inflammatory responses culminating in sepsis, which profoundly disrupts hemodynamics and contributes to AKI. Sepsis-induced vasodilation leads to a reduction in effective circulating blood volume, triggering compensatory activation of the renin-angiotensin-aldosterone system (RAAS). This neurohormonal response aims to restore perfusion pressure but paradoxically induces renal vasoconstriction, particularly of the efferent arterioles, elevating glomerular capillary pressure while diminishing overall renal blood flow. The resultant imbalance causes ischemic injury to renal tubular cells due to hypoperfusion despite maintained or increased glomerular filtration pressure. Moreover, sepsis-associated myocardial depression and systemic hypotension exacerbate renal hypoperfusion, creating a vicious cycle of worsening renal ischemia and injury. In the context of IPA, the fungal infection can intensify systemic inflammation and endothelial dysfunction, further impairing vascular tone and microcirculatory flow. This hemodynamic instability is a critical factor in the pathogenesis of sepsis-associated AKI, as evidenced by clinical observations of high AKI incidence in IPA patients complicated by sepsis and respiratory failure requiring ICU management [36,37]. The interplay between systemic vasodilation, RAAS activation, and cardiac dysfunction underscores the complexity of renal perfusion deficits in IPA-induced sepsis, highlighting the need for careful hemodynamic monitoring and management to mitigate AKI risk [38].

Renal Microcirculatory Disorders and Oxygen Supply-Demand Imbalance

The renal microcirculation is particularly vulnerable to the inflammatory milieu induced by IPA-associated sepsis, where mediators such as nitric oxide (NO) and endothelin-1 disrupt endothelial integrity and vascular tone. Elevated NO levels, while initially vasodilatory, can increase microvascular permeability and contribute to capillary leak syndrome, leading to interstitial edema that impairs oxygen diffusion to renal tubular cells. Concurrently, endothelin-1 promotes vasoconstriction and endothelial dysfunction, exacerbating microvascular flow heterogeneity. These alterations compromise red blood cell deformability and oxygen delivery, precipitating a mismatch between oxygen supply and demand within the renal cortex and medulla. Under hypoxic conditions, renal tubular epithelial cells undergo metabolic reprogramming, shifting from oxidative phosphorylation to anaerobic glycolysis. This metabolic adaptation reduces ATP generation efficiency, impairing energy-dependent cellular functions and promoting tubular cell injury and apoptosis. Additionally, activation of coagulation pathways and platelet aggregation in sepsis fosters microthrombi formation within the renal microvasculature, leading to focal ischemic necrosis of tubules. Such microcirculatory thrombosis has been implicated in the pathogenesis of acute tubular necrosis observed in sepsis-associated AKI. The cumulative effect of endothelial dysfunction, impaired oxygen delivery, metabolic derangement, and microvascular thrombosis culminates in significant renal parenchymal damage in IPA patients with sepsis [37,39,40]. Understanding these microcirculatory disturbances is essential for developing targeted therapies aimed at preserving renal oxygenation and preventing irreversible injury.

The Double-Edged Sword Effect of Fluid Resuscitation on Kidney Injury

**Figure 3:**
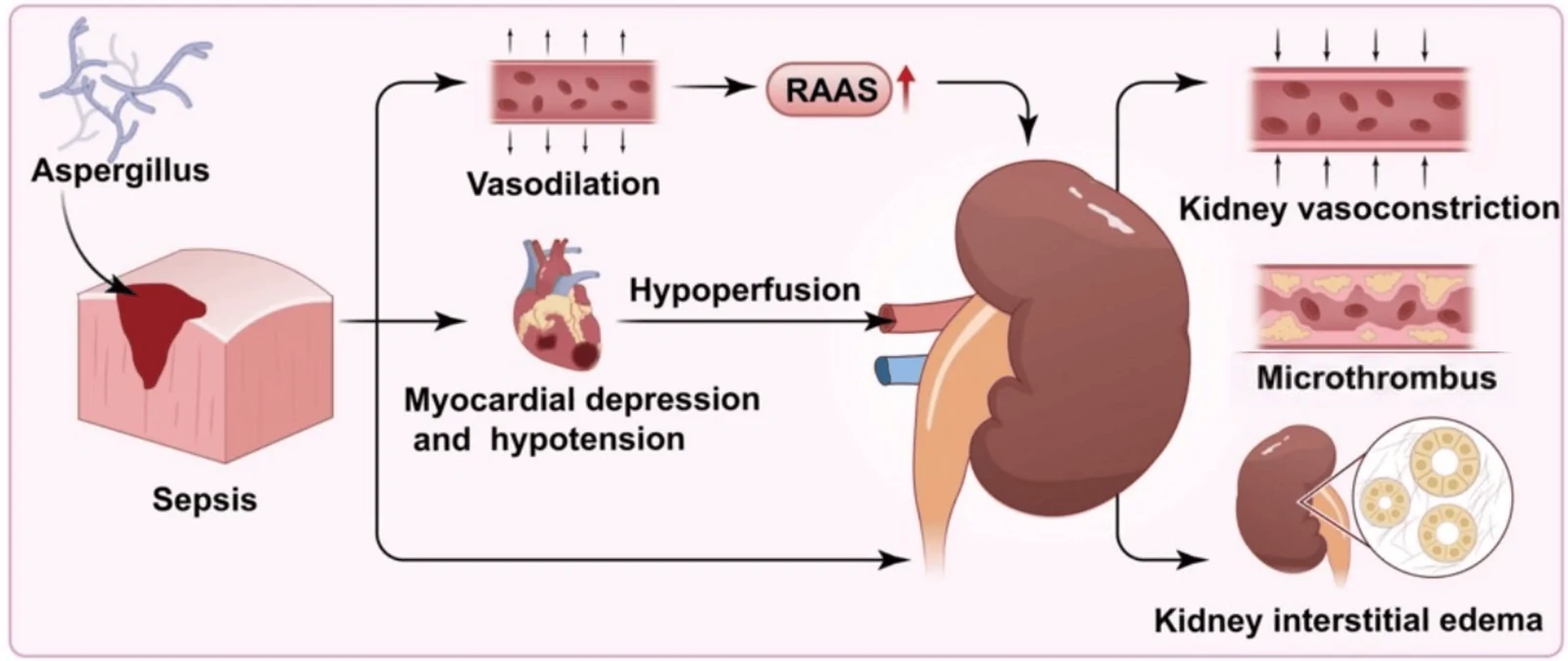
Hemodynamic derangements Sepsis induced by *Aspergillus* infection causes systemic vasodilation with activation of the RAAS, along with myocardial depression and hypotension, leading to reduced renal perfusion, renal vasoconstriction, and decreased renal blood flow. Sepsis also promotes renal microthrombus and interstitial edema, thereby aggravating renal damage. RAAS: renin-angiotensin-aldosterone system This figure was made using Adobe Illustrator (Adobe Inc., San Jose, CA, USA).

Fluid resuscitation remains a cornerstone in managing sepsis-induced hemodynamic instability in IPA patients; however, its impact on renal function is complex and potentially deleterious. Excessive fluid administration can elevate renal venous pressure and promote interstitial edema within the kidney, leading to increased intrarenal pressure that compresses renal tubules and microvasculature. This mechanical compression impairs tubular flow and exacerbates ischemia by restricting capillary perfusion, thereby aggravating AKI. Moreover, the use of synthetic colloids such as hydroxyethyl starch (HES) in fluid resuscitation has been associated with direct nephrotoxicity. HES molecules can accumulate within renal tubular epithelial cells, inducing osmotic nephrosis characterized by cellular swelling and dysfunction. Clinical studies have linked HES administration to increased incidence of AKI and requirement for renal replacement therapy in critically ill patients, underscoring the risks associated with these agents. Conversely, goal-directed fluid management strategies, emphasizing restrictive fluid administration tailored to individual hemodynamic parameters, have demonstrated potential renal protective effects in patients with IPA complicated by AKI. Such approaches aim to optimize effective circulating volume without precipitating fluid overload and renal congestion. Evidence suggests that restrictive fluid resuscitation may reduce renal interstitial edema and improve microcirculatory flow, thereby mitigating AKI severity. Nonetheless, the balance between adequate perfusion and fluid-induced renal injury remains delicate, necessitating vigilant monitoring and individualized therapy in IPA patients with sepsis-associated AKI [36,39]. Future research should focus on refining fluid resuscitation protocols to maximize renal protection while ensuring hemodynamic stability (Figure 3).

Multi-factor interaction and clinical translational research

Synergistic Effects of Fungal Toxins, Inflammation, and Drug Toxicity

The interplay between fungal toxins, host inflammatory responses, and antifungal drug toxicity significantly contributes to the pathogenesis of AKI in IPA. Experimental animal models have demonstrated that combined exposure to low-dose AmB and fungal toxins such as fungal toxins markedly exacerbates renal tubular necrosis compared to monotherapy, indicating a synergistic nephrotoxic effect. This synergy likely arises from the potentiation of cellular injury pathways, where fungal toxins sensitize renal tubular cells to drug-induced damage. Inflammatory mediators, particularly TNF-α, further amplify this effect by upregulating renal tubular cell receptors such as organic anion transporter 1 (OAT1), which facilitates increased uptake and intracellular accumulation of fungal toxins [41,42]. This receptor-mediated enhancement of toxin load within tubular cells intensifies cytotoxicity and promotes apoptosis. Concurrently, antifungal drugs like AmB induce oxidative stress, which exacerbates mitochondrial dysfunction and activates pro-apoptotic signaling cascades, thereby reinforcing the cytotoxic effects of fungal toxins. This “second hit” or “two-hit” model underscores the complex pathophysiology where drug-induced oxidative injury and toxin-mediated cellular stress converge, leading to amplified renal tubular damage. Clinically, this synergism manifests as more severe AKI in IPA patients receiving nephrotoxic antifungals, necessitating careful balancing of antifungal efficacy against renal safety. Understanding these interactions is critical for developing therapeutic strategies that mitigate renal injury, such as antioxidant co-therapy or modulation of inflammatory pathways, to improve outcomes in IPA complicated by AKI [12,34].

Host Genetic Susceptibility and Risk of Acute Kidney Injury

Genetic predisposition plays a pivotal role in modulating the risk and severity of AKI in patients with IPA. Single-nucleotide polymorphisms (SNPs) in key immune regulatory genes have been implicated in altering host susceptibility. Notably, the TNF-α promoter polymorphism at position -308G/A has been associated with increased AKI risk in IPA patients, likely due to its influence on TNF-α expression levels and subsequent inflammatory responses that exacerbate renal injury. Variations in complement component genes, particularly C5, modulate the generation of the potent inflammatory mediator C5a, which regulates neutrophil recruitment and activation within the kidney. Polymorphisms affecting C5a production can therefore influence the extent of tubular inflammation and damage during fungal infection. Furthermore, genetic polymorphisms in drug-metabolizing enzymes such as cytochrome P450 2C19 (CYP2C19) significantly impact voriconazole pharmacokinetics, leading to variable plasma drug concentrations. Poor metabolizers with certain CYP2C19 alleles exhibit elevated voriconazole levels, increasing the risk of nephrotoxicity, while rapid metabolizers may have subtherapeutic levels, compromising antifungal efficacy [12,43,44]. These pharmacogenetic factors necessitate individualized dosing strategies to optimize therapeutic outcomes and minimize renal toxicity. Collectively, these genetic determinants underscore the importance of integrating host genomics into risk stratification and personalized management of IPA-associated AKI, potentially guiding prophylactic interventions and tailored antifungal therapy.

Biomarkers and Early Warning Strategies

Early detection of AKI in IPA patients is crucial for timely intervention and improved prognosis. Emerging biomarkers such as kidney injury molecule-1 (KIM-1) and neutrophil gelatinase-associated lipocalin (NGAL) have demonstrated elevated urinary levels in IPA patients preceding changes in conventional serum creatinine, offering sensitive indicators of subclinical tubular injury. These markers reflect early tubular epithelial cell stress and damage, enabling prompt recognition of renal involvement. Additionally, plasma markers of NETs, including citrullinated histone H3, correlate with inflammatory activation and have been proposed as predictive indicators of AKI development. The detection of fungal toxin metabolites, such as rhizoxin, in plasma further complements the biomarker panel by indicating fungal burden and toxin-mediated renal insult. Integrative diagnostic models combining multiple biomarkers, for instance KIM-1, NGAL, and TNF-α, enhance sensitivity and specificity for early AKI diagnosis, surpassing single-marker approaches [12,45,46]. These multi-marker algorithms facilitate risk stratification and monitoring of disease progression, enabling clinicians to initiate renal protective measures and adjust antifungal regimens proactively. The incorporation of such biomarker-based early warning systems into clinical practice holds promise for reducing AKI incidence and improving outcomes in IPA patients.

Advances in targeted intervention strategies

Recent research has focused on targeted interventions to mitigate AKI in IPA by addressing oxidative stress, inflammation, and fungal toxicity. Antioxidants such as N-acetylcysteine (NAC) have shown efficacy in animal models by scavenging ROS generated during AmB and fungal toxin exposure, thereby attenuating oxidative damage and preserving renal tubular integrity. Complement pathway modulation represents another promising avenue; C5a receptor antagonists like PMX53 reduce complement-mediated inflammation and tubular apoptosis, as demonstrated in preclinical studies, suggesting potential for clinical translation. The development of novel antifungal agents with reduced nephrotoxicity, exemplified by rezafungin, offers therapeutic alternatives that maintain antifungal potency while minimizing renal adverse effects. Rezafungin’s favorable safety profile is currently under clinical evaluation, aiming to expand treatment options for IPA patients at risk of AKI [12,34,47].

Construction of multidisciplinary integrated management models

Effective management of IPA complicated by AKI necessitates a multidisciplinary approach integrating early warning systems, individualized antifungal therapy, and renal supportive care. Establishing early warning protocols that synthesize clinical data, imaging findings, and biomarker profiles enables risk stratification and timely intervention. Personalized antifungal regimens tailored to renal function, informed by pharmacogenetic testing and therapeutic drug monitoring, minimize nephrotoxicity while ensuring adequate antifungal exposure. Avoidance of concurrent nephrotoxic agents and dose adjustments based on renal clearance are critical components. Renal replacement therapies, particularly continuous renal replacement therapy (CRRT), offer advantages in clearing inflammatory mediators and fungal toxins, potentially mitigating renal injury and systemic inflammation. However, the optimal timing and modality of CRRT require further investigation. Collaborative care involving infectious disease specialists, nephrologists, intensivists, and pharmacists facilitates comprehensive management addressing the complex interplay of fungal infection, drug toxicity, and renal dysfunction. This integrated model aims to improve survival and renal outcomes in IPA patients with AKI by combining early detection, targeted therapy, and supportive interventions [12,34,44].

## Conclusions

The pathogenesis of AKI complicating IPA in adults is multifactorial, involving direct nephrotoxicity from fungal toxins (e.g., gliotoxin-induced apoptosis and oxidative stress), dysregulated host immune responses (excessive NETosis and complement activation), antifungal drug toxicity (notably amphotericin B), and hemodynamic disturbances leading to renal hypoperfusion. These factors interact synergistically, amplifying renal injury. 

From a clinical perspective, balancing effective antifungal therapy with renal safety is critical. Emerging agents such as rezafungin and isavuconazole offer improved nephrotoxicity profiles, while individualized dosing guided by therapeutic drug monitoring can mitigate drug-induced injury. Optimizing hemodynamic management and fluid resuscitation strategies is equally essential to preserve renal perfusion. Future research should focus on elucidating the molecular interplay among these pathways, validating sensitive biomarkers (e.g., KIM-1, NGAL) for early AKI detection, and developing targeted interventions, including fungal toxin neutralization, complement modulation, and antioxidant therapies. A multidisciplinary, individualized approach integrating early warning systems and renal supportive care holds promise for improving outcomes in IPA patients with AKI.
